# Inhaled nitric oxide: can it serve as a savior for COVID-19 and related respiratory and cardiovascular diseases?

**DOI:** 10.3389/fmicb.2023.1277552

**Published:** 2023-10-02

**Authors:** Yifan Zhao, Cheng Li, Shuai Zhang, Jiayu Cheng, Yucheng Liu, Xiaorong Han, Yinghui Wang, Yonggang Wang

**Affiliations:** ^1^Department of Cardiovascular Center, The First Hospital of Jilin University, Changchun, China; ^2^Department of Family and Community Medicine, Feinberg School of Medicine, McGaw Medical Center of Northwestern University, Chicago, IL, United States; ^3^Department of Special Care Center, Fuwai Hospital, National Clinical Research Center for Cardiovascular Diseases, National Center for Cardiovascular Diseases, Chinese Academy of Medical Science and Peking Union Medical College, Beijing, China

**Keywords:** nitric oxide, inhaled nitric oxide, COVID-19, pulmonary arterial hypertension, lung infection, acute respiratory distress syndrome

## Abstract

Nitric oxide (NO), as an important gaseous medium, plays a pivotal role in the human body, such as maintaining vascular homeostasis, regulating immune-inflammatory responses, inhibiting platelet aggregation, and inhibiting leukocyte adhesion. In recent years, the rapid prevalence of coronavirus disease 2019 (COVID-19) has greatly affected the daily lives and physical and mental health of people all over the world, and the therapeutic efficacy and resuscitation strategies for critically ill patients need to be further improved and perfected. Inhaled nitric oxide (iNO) is a selective pulmonary vasodilator, and some studies have demonstrated its potential therapeutic use for COVID-19, severe respiratory distress syndrome, pulmonary infections, and pulmonary hypertension. In this article, we describe the biochemistry and basic characteristics of NO and discuss whether iNO can act as a “savior” for COVID-19 and related respiratory and cardiovascular disorders to exert a potent clinical protective effect.

## 1. Introduction

Nitric oxide (NO) is an important pleiotropic regulator that is enzymatically synthesized *in vivo* from L-arginine by nitric oxide synthase (NOS), which precisely regulates cardiovascular, respiratory, neurological, and other multi-systems as well as a wide range of life activities by sending signals to specific targets. NOS has three different subtypes, namely endothelial nitric oxide synthase (eNOS), neuronal nitric oxide synthase (nNOS), and inducible nitric oxide synthase (iNOS), and the resulting NO can perform a variety of biological functions *in vivo*, such as vasodilatation, metabolic regulation, host defense, neurotransmitters, and so on (Mac Micking et al., 1997; Kashiwagi et al., 2023; Soundararajan et al., 2023). The coronavirus disease 2019 (COVID-19) has swept over the globe in recent years, with far-reaching consequences for people’s physical and mental health as well as the global social economy. When the human body is infected with severe acute respiratory syndrome coronavirus 2 (SARS-CoV-2), several clinical symptoms and pathologic features might arise, and some patients may continue to have some chronic sequelae after recovery (Chen et al., 2021; Yong, 2021). Inhaled nitric oxide (iNO), a therapeutic agent that delivers NO, has been approved for the treatment of neonatal pulmonary hypertension, and its role as an unconventional treatment with improved oxygenation and selective pulmonary vasodilatation appears to offer a promising therapeutic option for critically ill patients with COVID-19 (Kamenshchikov et al., 2022; Shei and Baranauskas, 2022). In addition to this, encouraging clinical benefits have been shown for cardiopulmonary diseases such as acute respiratory distress syndrome, lung infections, and pulmonary hypertension in adults (Sokol et al., 2016; Lisi et al., 2021). In this review, we present the sources and biological functions of NO, describe whether iNO can provide safe and effective therapeutic effects in the context of COVID-19, and discuss clinical applications in COVID-19-related cardiovascular and respiratory diseases.

## 2. The biosynthesis, biological functions, and therapeutic uses of NO

Because of its uncharged lipophilic feature, NO, as a key gas molecule in the living system, can freely travel between cells through the cell membrane and assist in the regulation of numerous physiological functions in the human body (Cinelli et al., 2020). Endogenous nitric oxide is created by macrophages, neurons, vascular smooth muscle, and cardiomyocytes via nitric oxide synthase’s conversion of L-arginine to L-citrulline (Nasyrova et al., 2020; Burov et al., 2022). Nitric oxide synthase has three isoforms. eNOS is normally expressed in vascular endothelial cells, and its mediated production of NO can diffuse into smooth muscle cells, activate soluble guanylate cyclase (sGC) to produce cyclic guanosine monophosphate (cGMP), and activate protein kinase G (PKG), which phosphorylates myosin light chain kinase (MLCK), thereby relaxing the smooth muscle and leading to vasodilation (Arnold et al., 1977; Ataei Ataabadi et al., 2020; Ma et al., 2023). In addition, NO produced by eNOS has physiological functions such as inhibition of platelet aggregation and adhesion, inhibition of leukocyte adhesion and vascular inflammation, inhibition of smooth muscle cell proliferation, anti-atherosclerosis, etc., which are important for maintaining the normal function of the cardiovascular system (Förstermann and Sessa, 2012; Li et al., 2014; Russo et al., 2023). nNOS regulates neurotransmitter release via the NO-NOsCG-cGMP signaling pathway and is also involved in the regulation of sympathetic nerves. When numerous pathologic causes cause a decrease in NO generation by nNOS, illnesses such as heart failure, hypertension, and renal insufficiency proceed (Sharma and Patel, 2017; Lundberg and Weitzberg, 2022). Cytokines, bacterial products, and other substances act as inducers to induce the release of large amounts of NO, i.e., inducible NOS (iNOS), from nitric oxide synthase in cells. iNOS is not often found in cells but is expressed in endothelial cells and immune cells after stimulation by certain inducers and exerts cytostatic or cytotoxic effects on tumor cells, microorganisms, or parasites. It is important to note that when NO is overexpressed, it can also have deleterious effects on normal tissues, such as infectious shock, pain, cancer, diabetes, etc. (Förstermann and Sessa, 2012; Cinelli et al., 2020; Lundberg and Weitzberg, 2022).

**Figure 1:**
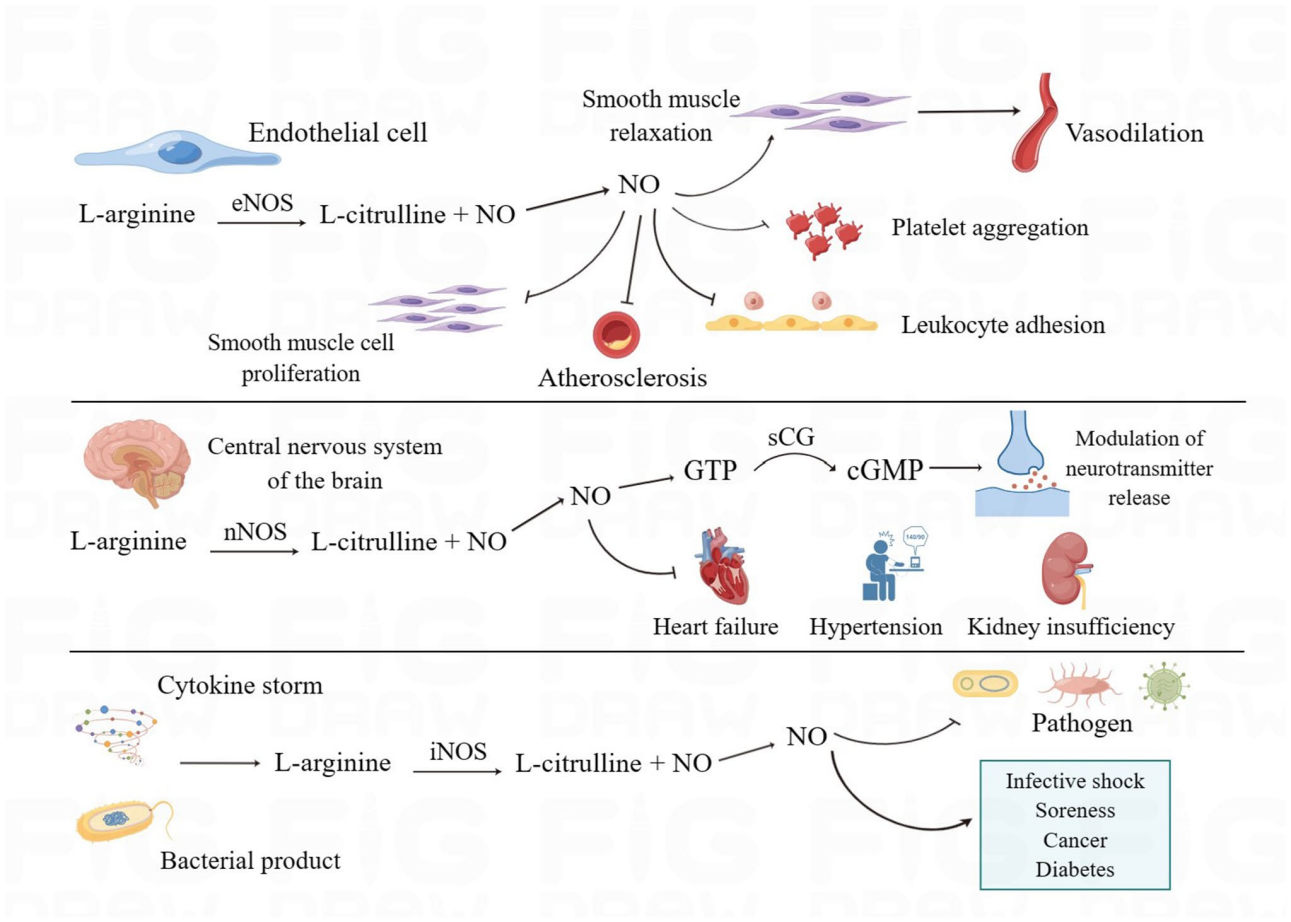
The biosynthesis and biological functions of NO. eNOS, nNOS, and iNOS catalyze the production of NO through L-arginine and perform different biological functions in the human body. eNOS mediates vasodilatation and inhibits platelet aggregation, leukocyte adhesion, smooth muscle cell proliferation, and atherosclerosis. nNOS, in addition to regulating the release of neurotransmitters, increases the development of cardiac and renal insufficiency and hypertension when NO production decreases. iNOS can produce large amounts of NO when stimulated, which on the one hand helps to enhance the ability of antibacterial and inflammatory responses and on the other hand can have adverse effects on normal tissues. (By Figdraw).

With the NO-NOsCG-cGMP signaling pathway playing a significant role, NO is a key signaling molecule for the control of vascular function, neurotransmission, the immune-inflammatory response, and other physiological activities. Through this signaling system, scientists are attempting to investigate treatment approaches for respiratory and cardiovascular illnesses. In addition to nitroglycerin and nitroprusside, which are known to be important NO donors for the treatment of angina pectoris and hypertensive crises (Miller and Megson, 2007), measurement of exhaled NO in a single breath can be used to monitor the treatment of allergic asthma (Rupani and Kent, 2022), and sGC activators (e.g., Riociguat, Vericiguat) and PDF5 inhibitors (e.g., sildenafil), which can synergize with endogenous NO, have shown promising therapeutic effects in the treatment of pulmonary hypertension (Lundberg and Weitzberg, 2022; Grześk et al., 2023). Since NO is highly reactive and easily passes through biological membranes, researchers have attempted to explore strategies to enhance NO release and exogenous NO supplementation. Roberts et al. discovered that iNO not only diffuses through the lungs but at the same time selectively dilates the pulmonary vasculature, innovatively applying NO to clinical practice (Roberts et al., 1992). Since 1993, iNO has been extensively studied as one of the potential treatments for acute respiratory distress syndrome (ARDS) and was approved by the FDA in 1999 for the treatment of persistent pulmonary hypertension in neonates (American Academy of Pediatrics, 2000; Redaelli et al., 2023). iNO is potentially beneficial in reducing right heart load and reducing hypoxemia due to ARDS when used in the low dose range (10–80 ppm). Systemic vasodilatory effects were not demonstrated at inhalation concentrations up to 160 ppm. At high doses (>160 ppm), iNO kills a wide range of pathogens, including bacteria, fungi, and viruses, and its antimicrobial activity is enhanced when used in combination with antibiotics (Barnes and Brisbois, 2020; Sorbo et al., 2022). Additionally, iNO is advantageous for myocardial damage, cardiac arrest, neuroprotection, recovering organ function following organ transplantation, and patient survival (Barnes and Brisbois, 2020; Redaelli et al., 2022). In this article, we focus on the possible therapeutic potential of iNO in COVID-19, focusing on its clinical applications and therapeutic perspectives in the respiratory and cardiovascular systems (Figure 1).

## 3. Clinical application of iNO in COVID-19

### 3.1. The role of NO in COVID-19

SARS-CoV-2 is the pathogen responsible for COVID-19. When it infects a person, it can cause structural and functional damage to a number of organs, with the respiratory system being the primary manifestation. Affected individuals may experience no symptoms, mild symptoms, severe illness, or even death (Guimarães et al., 2021; Stratton et al., 2021). Infection with SARS-CoV-2 can lead to decreased NO production and utilization by a number of mechanisms, including direct infection of endothelial cells, amplification of the detrimental effects of the angiotensin-converting enzyme (ACE)/angiotensin II (AngII)/angiotensin II type 1 (AT1) axis following down-regulation of angiotensin-converting enzyme 2 (ACE2) expression, and further development of the hyperinflammatory state as a cytokine storm (Dominic et al., 2021; Fang et al., 2021; Ferrari and Protti, 2022; Montiel et al., 2022). On the other hand, studies have demonstrated that NO has direct or indirect antiviral effects (Lisi et al., 2021). The inflammatory response caused by viral infection activates macrophages with increased expression of iNOS and the release of large amounts of NO, which plays a role in regulating host immune function against pathogens (Guimarães et al., 2021). SARS-CoV-2 can infect the nervous system and damage the blood–brain barrier via binding to ACE2 in vascular smooth muscle cells and brain capillary endothelial cells. Large levels of NO are consequently created to prevent viral replication after iNOS is triggered. It is important to keep in mind, though, that excessive NO-related free radical generation might harm the brain, leaving some patients with neurological deficiencies like sleep difficulties (Cespuglio et al., 2021). In addition, NO inhibits or impairs SARS-CoV-2 by acting on the viral spiking protein and the cysteine protease encoded by SARS-CoV-2 Orf1a (Stefano et al., 2020). The antiviral effect of NO was similarly demonstrated in a study by Akaberi et al. The viral inhibition following release of NO from the NO-donor nitroso-N-acetylpenicillamine (SNAP) was dose-dependent, with a delayed or blocking effect on the occurrence of cytopathic effects (CPE) in virus-infected cells. The reduction in viral replication may be related to the nitrosylation of the SARS-CoV-2 3CL cysteine protease, although this has to be further validated (Akaberi et al., 2020). The antiviral capacity of NO was further explored in a clinical study. The patients with mild COVID-19 infection were randomly assigned to a nitric oxide nasal spray (NONS) treatment group and a placebo group, and the results demonstrated that NONS can play a clinically beneficial role by effectively and safely reducing the RNA load of SARS-CoV-2 and decreasing further virus transmission (Winchester et al., 2021). However, an *in vitro* experiment conducted by Rousseaud’s team negated the antiviral properties of gaseous NO. No effect on viral load was observed after exposing SARS-CoV-2-infected cells to high doses of gaseous NO (Rousseaud et al., 2023). In addition to this, the massive production of NO can cause tissue and cell death, and the cytotoxic effects of reactive oxygen and nitrogen species can be damaging to the body (Lisi et al., 2021). Therefore, further investigation and refinement are needed regarding the antiviral treatment and clinical safety of NO.

SARS-CoV-2 is a respiratory pathogen that can damage the respiratory system, resulting in severe hypoxemia and dyspnea; however, some patients present with silent hypoxia, i.e., severe hypoxemia without significant dyspnea (Yuki et al., 2020; Mandal, 2023). Mortaz et al. found that the NO content of erythrocytes in COVID-19 patients was higher than that in non-COVID-19 patients. When tissue hypoxia and oxidative stress occur *in vivo*, erythrocytes produce NO through S-nitrosoprotein (SNO-Hb) transfer and erythrocyte nitric oxide synthase, which diastole the small blood vessels, regulate the local blood flow, and promote the release of oxygen into hypoxic tissues, thereby playing a role in the protection of COVID-19 patients suffering from silent hypoxia (Mortaz et al., 2020; Mandal, 2023). It has been shown that patients with severe COVID-19 have reduced HbNO compared to non-COVID-19 patients with similar cardiovascular risk, which correlates with reduced NO bioavailability and is a potential specific biomarker of endothelial dysfunction (Ferrari and Protti, 2022). The results, however, were disputed. Nogueira et al. came to the conclusion that endogenous nitrite is influenced by outside factors such as diet, lifestyle, and oral flora and that treatment of the blood with N-acetylcysteine and ascorbic acid artificially increases HbNO when endogenous nitrite is present. This means that HbNO does not reflect endothelial function at an early stage and that further assay method optimization is required (Nogueira et al., 2022).

The COVID-19 pandemic has placed a significant burden on healthcare systems around the world, and the rational allocation and utilization of healthcare resources and the economy in response to the severity of the disease is an urgent issue, so researchers are looking for a convenient, safe, and non-invasive biomarker to stratify the risk level of the disease. Fraction of exhaled nitric oxide (FeNO) is a simple, reproducible, non-invasive biomarker with diagnostic value for nonspecific respiratory symptoms and airway inflammation and is used as an adjunctive assessment for the diagnosis of asthma, predicting the response to inhaled corticosteroids, and guiding clinical treatment (Guida et al., 2023; Murugesan et al., 2023). FeNO levels in COVID-19 patients and the clinical value of FeNO in COVID-19 are controversial. On the one hand, SARS-CoV-2 infection induces an inflammatory response with a cytokine storm that induces NO production by iNOS (Karki et al., 2021), and on the other hand, it was shown that the severity of SARS-CoV-2 infection was negatively correlated with FeNO production, which was related to the fact that ACE2 attenuated the production of NO by iNOS in airway epithelial cells, whereas high expression of ACE2 had a stronger susceptibility to SARS-CoV-2 (Betancor et al., 2022a). It has been suggested that FeNO levels are elevated in COVID-19 patients compared to healthy individuals. Kerget et al. concluded that FeNO levels can be used to assess lung parenchymal involvement in COVID-19 patients and that patients with cytokine storms have higher levels of FeNO (Kerget et al., 2022). Whereas the results of a single-center prospective study showed that the more severe the disease, the lower the FeNO levels in those with COVID-19, with patients with measurements ≤ 11.8 PPB having worse clinical outcomes (Lior et al., 2022). It has been questioned whether FeNO levels in COVID-19 patients can be used to predict the severity and prognosis of the disease. Betancor et al. demonstrated that FeNO levels were normal during the acute phase of SARS-CoV-2 infection and increased slightly during the recovery phase, independently of disease severity (Betancor et al., 2022a,b). Even in the group with post-COVID-19 syndrome, FeNO does not have a clear clinical value (Maniscalco et al., 2022). Age, gender, smoking, concurrent respiratory conditions, and whether or not the patient has had corticosteroid therapy are some of the variables that can affect FeNO levels. The sample size and the FeNO instruments also have an impact on the study’s outcome (Betancor et al., 2022a). Therefore, it is unknown whether FeNO truly has potential therapeutic value for COVID-19 disease risk categorization and treatment regimen advice. Multicenter, large-sample trials are still required to make this determination.

### 3.2. Clinical application of iNO in COVID-19

Numerous studies have investigated and talked about the clinical use of iNO in COVID-19. The idea that iNO can improve oxygenation to varying degrees in this subset of patients and lessen the use of invasive respiratory support techniques is supported by a number of pieces of evidence, despite the fact that iNO cannot be used as a conventional treatment for refractory hypoxemia and ARDS brought on by COVID-19 and does not significantly improve mortality or prognosis (Parikh et al., 2020; Garfield et al., 2021; Lotz et al., 2021; Safaee Fakhr et al., 2021; Al Sulaiman et al., 2022). It has been suggested that appropriate iNO therapy given in the early stages of COVID-19 infection may delay disease progression, especially in patients who are older or have multiple comorbidities that result in decreased endogenous nitric oxide production (Adusumilli et al., 2020). There is even proof that pregnant individuals with severe or critical COVID-19 benefit from high-dose iNO therapy (Safaee Fakhr et al., 2020).

Smoking puts human health in danger, raises the prevalence of respiratory and cardiovascular disorders, and lowers life expectancy rates (Ambrose and Barua, 2004; Lugg et al., 2022). However, according to other research, smoking appears to protect against COVID-19 and reduce SARS-CoV-2 infection, which is known as the smoker’s paradox (Usman et al., 2021; Papadopoulos et al., 2023). Epidemiologic studies have found that only a small proportion of patients in the smoking population, which makes up a relatively large proportion of the smoking population in several countries around the world, have COVID-19 (Berlin et al., 2020; Hedenstierna et al., 2020; Usman et al., 2021). Although the idea that smoking has a protective effect may seem naive, a partially plausible explanation exists from a pathophysiological point of view:1. It has been suggested that the large elevation of NO in the lower respiratory tract after smoking, present in the epithelial lining fluid, leads to an increase in the reactivity of a bioequivalent form with a similar bioactivity to that of NO, thus protecting against SARS-CoV-2 infection (Chambers et al., 1998; Zhao et al., 2015). 2. Tobacco smoke may enhance the uncoupling of eNOS and promote transcription, leading to increased NO production (Papadopoulos et al., 2023). 3. CYP450 in the liver mediates NO release from nitrate when the typical eNOS pathway is impaired (Papadopoulos et al., 2023). 4. The anti-inflammatory effects of nicotine and the suppression of systemic cytokines in smokers help to attenuate the cytokine storm of COVID-19 (Wang et al., 2003; Garufi et al., 2020; Usman et al., 2021). 5. Smoking may upregulate ACE2 expression and thus reduce disease severity (Usman et al., 2021). Hedenstierna et al. suggested that short, high doses of iNO have a preventive effect on COVID-19. This is because cigarettes contain a high amount of NO, and a high concentration of NO during a single inhalation can quickly reach the virus by diffusion. As a result, COVID-19 prophylaxis may be possible with high-dose, intermittent delivery of iNO over a short period of time. But smoking is undeniably a risk factor for COVID-19, and continuing to smoke over time raises the likelihood of contracting the virus (Hedenstierna et al., 2020).

Regarding the clinical effectiveness and safety of iNO, there is still some disagreement. Because COVID-19-induced ARDS has more significant vascular endothelial damage and microthrombosis than non-COVID-19-induced ARDS, in addition to diffuse loss of pulmonary vasoconstriction and peri-alveolar solidity, which may affect iNO’s ability to improve oxygenation (Longobardo et al., 2021; Bonizzoli et al., 2022). In terms of safety, Lotz et al. found that iNO appears to be associated with an increase in acute kidney injury with hospital-acquired pneumonia (Al Sulaiman et al., 2022). iNO therapy is an innovative tool in COVID-19, but due to the complexity of the pathogenesis of COVID-19 and the variety of clinical symptoms, the patient population, timing, dosage, and mode of administration of iNO therapy are still unknown, and these are the difficulties that need to be overcome in future studies (Frostell and Hedenstierna, 2021; Shei and Baranauskas, 2022).

## 4. Application of iNO in respiratory and cardiovascular diseases

### 4.1. Acute respiratory distress syndrome

Acute respiratory distress syndrome (ARDS) is caused by various pathogenic factors that damage alveolar epithelial cells and capillary endothelial cells, resulting in pulmonary edema and severe hypoxemia, thus causing acute respiratory distress (Meyer et al., 2021; Bos and Ware, 2022). Previous research has concluded that iNO is a valuable therapeutic option for severe hypoxemia caused by ARDS because of its ability to selectively dilate the pulmonary vasculature and improve the ventilation-to-blood flow ratio, and that iNO’s therapeutic efficacy can be improved when used in combination with other therapeutic means (Kaisers et al., 2003). Several studies, however, have indicated that iNO only increases the oxygenation index temporarily, does not improve long-term patient survival appreciably, and may even increase the risk of mortality and renal impairment (Adhikari et al., 2007; Afshari et al., 2011; Gebistorf et al., 2016). As a result, iNO is not indicated as a first-line treatment for ARDS (Gebistorf et al., 2016).

However, it is worth noting that iNO seems to have a wider use in patients with COVID-19-associated acute respiratory distress syndrome (C-ARDS; Mekontso Dessap et al., 2023). For persistent refractory hypoxemia due to COVID-19, inhaled 5–20 ppm NO appears to be beneficial in improving oxygenation in addition to neuromuscular blockade and optimizing positive end-expiratory pressure therapy (Matthay et al., 2020). The results of a multicenter retrospective study also confirmed the clinical value of iNO, with nearly half of the patients having a better oxygenation response after iNO, and about half of the patients who met the indications for ECMO no longer needed to be dependent on ECMO after iNO (51.6%), and this group of patients who responded well to iNO had a better prognosis than those who still met the indications for ECMO after iNO (Mekontso Dessap et al., 2023). However, as mentioned earlier, the role of iNO in improving hypoxemia remains controversial, which is related to the complex pathogenesis of severe hypoxemia in patients with C-ARDS (Archer et al., 2020; Bonizzoli et al., 2022; Mekontso Dessap et al., 2023). In addition to this, the safety of iNO and whether it is synergistic with other therapeutic measures are not yet known; therefore, further research and exploration are needed.

### 4.2. Lung infections

Antibiotic misuse can result in the growth of bacteria that are multidrug resistant, which can prolong hospital stays, necessitate the use of stronger drugs, raise their dosage, and cause complications from various infections that increase the likelihood of negative clinical outcomes (Santacroce et al., 2023; Yao et al., 2023). Contrary to conventional treatments, iNO is not linked to drug resistance, and because it diffuses quickly across biological fluids and membranes, NO can get into places where intravenous medications cannot. Because it may efficiently suppress or kill germs, iNO has great therapeutic potential in the field of anti-infective therapy (Sorbo et al., 2022).

*In vitro* studies have demonstrated that the antimicrobial capacity of iNO is dose-dependent. Low doses of iNO less than 160 ppm have only an inhibitory effect on bacteria, but doses greater than 160 ppm can kill bacterial colonies of *Escherichia coli*, *Pseudomonas aeruginosa*, and *Staphylococcus aureus*, and when applied at higher doses (200 ppm), it has a significant bactericidal effect on multidrug-resistant cocci (Ghaffari et al., 2006; Sorbo et al., 2022). It is unavoidable that NO can bind to hemoglobin and generate methemoglobin when it is injected into the body in large concentrations, interfering with normal oxygen transport even if *in vitro* studies have strongly supported the antibacterial impact of iNO (Signori et al., 2022). High-dose, intermittent dosing therefore seems to be an effective solution. With such a solution in mind, researchers have conducted numerous *in vivo* studies. Several animal studies have shown that iNO has good antimicrobial activity and no side effects when the dose is well balanced with the duration of exposure, and even Michaelsen et al. have demonstrated the safety of high doses of continuous iNO in healthy animals (Miller et al., 2013; Michaelsen et al., 2021; Wiegand et al., 2021; Sorbo et al., 2022). Also, this regimen showed better clinical efficacy in clinical studies. The researchers showed no significant adverse events and a good safety profile after giving high doses of intermittent iNO to healthy individuals and patients with pulmonary infections (Miller et al., 2012; Sorbo et al., 2022). Flume et al. administered iNO to nine patients with nontuberculous mycobacterial lung disease (NTM-PD), four of whom tested negative on sputum culture after 3 weeks and had no safety concerns with the treatment. Although three of the patients were found to have positive sputum cultures after retesting 3 months after stopping treatment, the NTM load was lower than before (Flume et al., 2023). In a prospective double-blind randomized controlled study, infants with acute bronchiolitis were randomly assigned to a standard treatment group and to different iNO concentrations in combination with standard treatment. The results showed that high-dose intermittent injections of 150 ppm iNO were clinically more effective and well tolerated than the other groups (Goldbart et al., 2023). However, iNO has not shown bactericidal efficacy in clinical studies or *in vitro* experiments, and further exploration is needed to increase the dosage and prolong the exposure time to achieve complete eradication of pathogenic microorganisms while ensuring human safety (Deppisch et al., 2016; Sorbo et al., 2022).

### 4.3. Pulmonary arterial hypertension

Pulmonary hypertension (PH) is a life-threatening vascular disease in which persistent vasoconstriction, increased vessel wall stiffness, pulmonary vascular remodeling, and *in situ* thrombus can cause increased pulmonary vascular resistance (PVR), leading to increased pulmonary artery pressures, which can lead to progressive right heart insufficiency and heart failure (Keshavarz et al., 2020; Zolty, 2020). The disease has diverse etiologies, an insidious onset, and complex pathophysiologic mechanisms and is usually diagnosed when the patient presents with dyspnea, fatigue, and cyanosis of the lips and mouth (Keshavarz et al., 2020). Based on the pathogenesis of PH, a number of specific therapeutic options have been developed, including prostacyclin analogs (Montani et al., 2014), endothelin receptor antagonists (Goldberg et al., 2017), soluble guanylate cyclase agonists (Stasch et al., 2011), and phosphodiesterase inhibitors (Triposkiadis et al., 2022).

The NO-sCG-cGMP signaling pathway plays an important role in the pathogenesis of PH, and several targeted therapies have been developed based on this signaling pathway (Keshavarz et al., 2020). Studies have shown that NOS expression is reduced and NO bioavailability is decreased in patients with PH (Giaid and Saleh, 1995; Demoncheaux et al., 2005). iNO selectively lowers pulmonary arterial pressure without systemic hypotensive effects and improves mean pulmonary arterial pressure, pulmonary vascular resistance, and oxygenation in adult PH patients with severe hypoxemia and respiratory failure as a “rescue” therapy (Keshavarz et al., 2020). Feng et al. found that iNO improved oxygenation status while reducing the risk of right heart failure in COVID-19 patients with preexisting pulmonary hypertension (Feng et al., 2021). A clinical trial has shown that pulsed iNO improves daily physical activity in patients with pulmonary hypertension due to pulmonary fibrosis and is beneficial and safe in the clinical management of the disease (Nathan et al., 2020). In addition, for pulmonary hypertension after cardiac surgery, iNO not only improves hemodynamics, but also has a protective effect on systemic organ function (Nakane et al., 2021). However, iNO is currently only approved for the treatment of persistent pulmonary hypertension in neonates, and there is much controversy over adult pulmonary hypertension. For example, iNO has a short half-life, can produce toxic metabolites, has low outpatient penetration, is costly, and appears to have no clinical benefit in terms of prognosis for some patients (Sardo et al., 2018; Keshavarz et al., 2020; Redaelli et al., 2022). Therefore, although iNO is being considered as a therapeutic option for patients with severely impaired respiratory function, large randomized controlled trials are needed to further judge its clinical benefits and safety.

**Figure 2:**
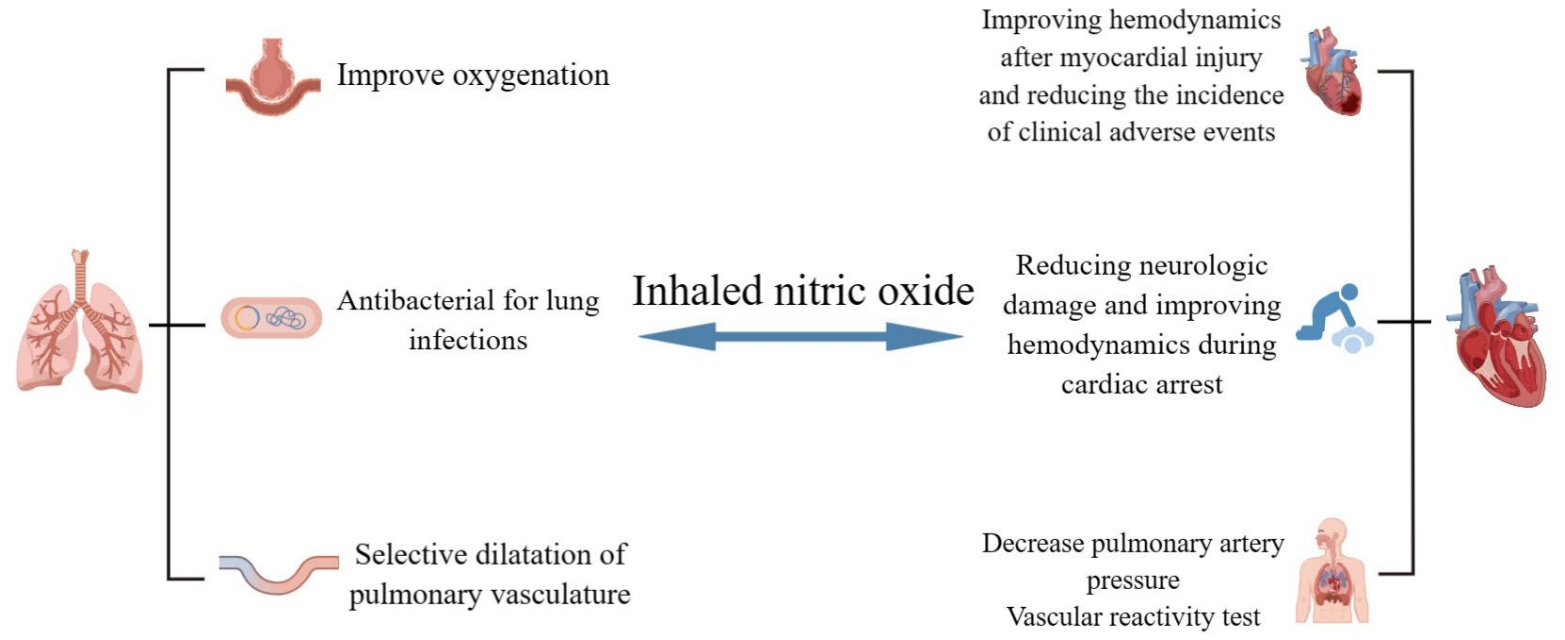
Potential clinical applications of iNO in respiratory and cardiovascular diseases. iNO can be used as a “rescue” or “innovative” therapy for COVID-19-related respiratory and cardiovascular diseases, with potential clinical benefits in hypoxemia, pulmonary infections, dilated pulmonary vessels, myocardial injury, cardiac arrest, and pulmonary hypertension. The potential clinical benefits of these therapies in hypoxemia However, high-quality evidence for these clinical applications is still lacking and needs to be explored and refined. (By Figdraw).

In addition to its therapeutic applications, the vascular reactivity test for iNO is used to identify patients with pulmonary hypertension in whom elevated pulmonary vascular resistance is not accompanied by severe vascular remodeling. The test is only used to determine whether calcium channel blockers are necessary for treatment in patients with idiopathic, hereditary, or drug-induced pulmonary hypertension, and there is a chance that giving iNO to patients with other types of pulmonary hypertension could cause pulmonary edema (Redaelli et al., 2022). iNO can be used to determine the response to calcium channel blockers in patients with pulmonary hypertension, and whether this test of vascular reactivity is indicative of patient prognosis was studied by Malhotra et al. The results of the study showed that a decrease in PVR and mPAP after iNO implies better long-term survival, is an independent predictor of survival, and facilitates risk stratification of patients with pulmonary hypertension, even in patients who are not suitable for treatment with calcium channel blockers (Malhotra et al., 2011). The clinical value of repeated iNO vascular reactivity testing in patients with pulmonary hypertension was first investigated by Tooba et al. The results showed that the reactivity of the pulmonary vasculature to iNO decreased over time, which may be related to disease progression leading to vasoconstrictive hypoplasia and to the impact of vasodilatory reserve by specific treatments for pulmonary arterial hypertension (Tooba et al., 2020). Ishii et al. confirmed Tooba’s findings on altered pulmonary vascular reactivity but differed from them by suggesting that the results of vascular reactivity testing after treatment of pulmonary hypertension could suggest prognostic information. Patients with reduced vascular reactivity had worse clinical outcomes than those with preserved vascular reactivity, which facilitates earlier identification of high-risk individuals in the follow-up of pulmonary hypertension and facilitates early intervention and treatment (Ishii et al., 2023; Figure 2).

## 5. Summary and outlook

NO, as a free radical gas, is involved in numerous life activities through direct interaction with target cells or the formation of multiple nitrogen oxides. The biochemistry and mediated signaling pathways of NO are complex and diverse, and therapeutic agents and treatments derived from them are widely used in clinical practice. iNO is a method of delivering nitric oxide as a selective pulmonary vasodilator with excellent therapeutic potential for COVID-19 and other related respiratory and cardiovascular diseases. It has also recently inspired the creation of portable NO generators, an area of research that is currently quite active. Even though iNO appears to be a rescue therapy for seriously ill patients, there are toxicity concerns that must be carefully considered, as they are driven by pathophysiological signals *in vivo*. Clinical studies on iNO have been somewhat successful, but there is still a long way to go before it becomes a mature and safe therapy.

## Author contributions

YZ: Writing – original draft. CL: Writing – review & editing. SZ: Writing – review & editing. JC: Writing – review & editing. YL: Writing – review & editing. XH: Writing – review & editing. YiW: Writing – review & editing. YoW: Writing – review & editing.
